# Obstetric Practice in China

**Published:** 1906-04

**Authors:** J. M. Oxner

**Affiliations:** Ping-tu, Shan-Tung, China


### Obstetric Practice in China.

PING-TU, SHAN-TUNG, CHINA, January 24, 1906.

*Texas Medical Journal, Austin, Texas, U. S. A.*

Thinking it might be of interest to some of the readers of the JOURNAL to see the report of a case or two from China, I wish to make the following report of two cases of obstetrics.

First, I wish to say, a Chinese is a queer compound, both by nature and makeup. It is absolutely against Chinese custom for a man to see a woman in labor, and more especially a foreigner. Then their power of endurance is beyond my conception.

CASE 1. A woman, 30 years of age, in her fifth confinement. I was called to see her on the sixth day of labor; found her lying on a k'ang,—a mud-brick bed,—with dogs, cats, pigs and chickens going in and out of the "lying-in chamber" at their pleasure. Just a few feet from the k'ang, in the same room was a manger, a grain mill, and a donkey busy grinding away. In short, it was the bed-room, the dining department, the kitchen, the dog quarters, the hog pen, the barn-yard, and the donkey stalls all in one for convenience. The patient had had no pains for two days, her pulse was very good, but her bowels had not moved for about a week; her bladder was very much distended. On examination, I found the child was dead—it was lying crosswise with one arm born and torn off at the shoulder; she had been in this condition for about three days. They (the old Chinese women) were trying to pull the child from her when this calamity occurred. There was no chance, and as the child was dead, no necessity of turning the child, but to take it away by pieces was the easiest way out. I sent my medical helper back to the dispensary for a lot of instruments, which I didn't have with me, and he came bringing tooth forceps, a tonsillotome, wire saws, a few test tubes, etc., but among them was the instrument I wanted. This shows how much even Chinese doctors know about instruments. I cut the child in two parts and took away one-half at a time; used creoline, carbolic acid, bichloride of mercury, soap and a rough towel as after treatment, locally, of course. Irrigated the womb the second and third days, and gave a little medicine internally; she was up in two and a half weeks.

CASE 2. As the first one, the child dead, one arm off, no pains. I did not have to cut the child away, but disemboweled it and took it away very easily. This case, however, has a vesicovaginal fistula as a result; otherwise she is well. They have promised that I might operate on her after their New Year.

For these two cases I realized about $5 gold for the dispensary. To practice in China's filth is enough to blush the moral modesty of a dignified germ doctor, and put him to open shame, but the opening is large—I have a million and a half in my territory.

Ping-tu, China. J. M. OXNER, M. D.

---

"THE 'Red Back' gets better the older it gets."—*B. S. Ezelle, Kosse.*

---

THE INHALATION CABINET (see advertisements) will be exhibited by Dr. Roberts, president of the company, at the annual meeting of the State Medical Association at Fort Worth, April 24-5-6. It is worth while to investigate its claims and value.

